# The clinical evolution of patients with idiopathic spinal cord herniation: a case series

**DOI:** 10.1038/s41394-024-00684-9

**Published:** 2024-10-09

**Authors:** Stéphanie Flageol, Sylvine Carrondo-Cottin, André Turmel, Isabelle Côté, Jérôme Paquet

**Affiliations:** 1 Physical Medicine and Rehabilitation, CHU de Québec – Université Laval, Québec City, QC Canada; 2 Department of Neurosciences, CHU de Québec – Université Laval Research Centre, Québec City, QC Canada; 3 Neurosurgery, CHU de Québec – Université Laval, Québec City, QC Canada; 4 Centre interdisciplinaire de recherche en réadaptation et intégration sociale (CIRRIS), Québec City, QC Canada

**Keywords:** Spinal cord diseases, Therapeutics

## Abstract

**Study design:**

Retrospective case series of 48 patients.

**Objectives:**

This study’s primary objective was to provide a clinical description of a group of individuals with a working diagnosis of idiopathic spinal cord herniation (ISCH). The secondary objectives were to appreciate the natural history of these patients and describe their clinical evolution with conservative or surgical management.

**Setting:**

The study was carried out at l’Hôpital de l’Enfant-Jésus, CHU de Québec (Québec, Canada), a tertiary care university hospital.

**Methods:**

This case series study is based on routinely collected data. Forty-eight (48) cases were identified as having an ISCH on MR imaging, between 2009 and 2019. Their medical files have been searched retrospectively. Patient characteristics were described according to their asymptomatic or symptomatic status.

**Results:**

The mean age of patients at the time of diagnosis was 52.5 years. Most of the patients identified were asymptomatic (69%) and followed clinically. The main neurologic presentation for the symptomatic group was Brown-Séquard-like syndrome. 20% of the symptomatic patients were rapidly treated surgically after consultation with the neurosurgeon. The mean follow-up duration was 56 months for asymptomatic patients and 51 months for symptomatic patients. Most of our patients (41 out of 45) were considered stable or unchanged at follow-up. There was no neurological progression in all asymptomatic patients.

**Conclusions:**

Our study shows that ISCH and its variants are not always symptomatic and may be a fortuitous finding. As the natural history may be non-progressive, it is probably appropriate to treat some cases expectantly.

## Introduction

Idiopathic spinal cord herniation (ISCH) is a rare disease, characterized by a ventral or ventrolateral herniation of the spinal cord through an anterior dural defect [1–3]. The pathophysiology of ISCH remains unclear. This mainly affects thoracic levels [1, 3, 4]. Relation to the physiologic kyphosis of the thoracic spine has been hypothesized, leading to an anterior displacement of the cord [2, 5, 6]. The usual presentation described is a progressive myelopathy [2], such as a Brown-Séquard-like syndrome or a spastic paraparesis [7, 8]. Symptoms are usually insidious and vague at onset, leading to a prolonged delay, from symptom onset to diagnosis [9].

High resolution phase-contrast MR imaging (MRI) is the radiologic gold standard to confirm ISCH. The key feature is an anterior displacement of the spinal cord, with absence of cerebrospinal fluid (CSF) flow ventral to the herniated cord and a normal CSF flow pattern dorsal to the cord, which helps to eliminate the presence of an arachnoid cyst [5].

The current literature mostly describes symptomatic cases and surgical management of ISCH. Some even argue that surgery should be the only treatment offered [8]. However, incidentally detected cases are becoming more prevalent with the increased access and quality of MRI imaging, which has also permitted to identify anterior spinal cord adhesion without herniation [2, 10]. It is still unclear if there is a radiologic continuum between spinal cord adhesion and herniation and if the presenting symptoms are similar.

No study in the literature was found reporting asymptomatic individuals with true spinal cord herniation. Only Lee et al. describe asymptomatic individuals with anterior spinal cord adhesion [10]. They identified 12 asymptomatic individuals with focal anterior displacement of the spinal cord without evidence of intradural mass or spinal cord herniation. Follow-up MRI obtained in 4 individuals showed no interval change and none showed symptoms of myelopathy during the follow-up period.

Because of the relative rarity of ISCH, little information is available about the natural history of this condition. Only few studies with small samples describe the conservative management of such individuals [4, 10–12]. It was therefore necessary to evaluate and study the different presentations to try to clarify the pathophysiology and the possible clinical and radiological continuum of this entity.

The primary objective of this study was to provide a clinical description of a group of individuals with a working diagnosis of ISCH. The secondary objectives were to describe the natural history of these patients and report the clinical and neurologic evolution with conservative or surgical management.

## Methods

The study design is a case series study based on routinely collected data from individuals with a working diagnosis of ISCH (including anterior spinal cord adhesion) determined on MRI by the neurosurgical department of CHU de Québec, a tertiary neurosurgical center based in Quebec City, Canada.

**Fig. 1:**
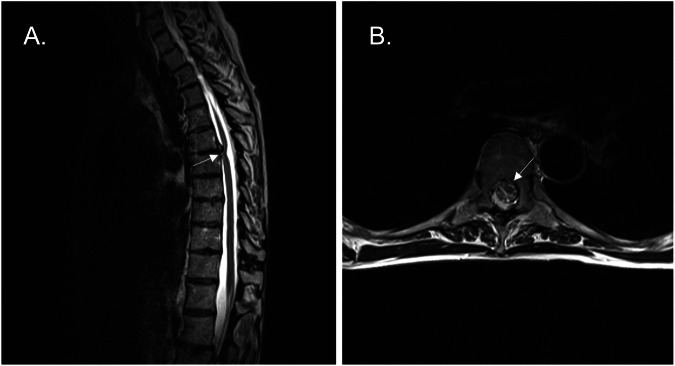
MRI featuring ISCH. **A** Sagittal and **B** axial MRI demonstrate sharp anterior angulation or the thoracic spinal cord with absence of CSF ventrally and widening of the subarachnoid space dorsally.

Included individuals were 18 years or older and were diagnosed with ISCH by thoracic spine MRI, as requested by primary care physicians for diverse indications. All MRI were read by trained neuroradiologists and reviewed by a trained spine neurosurgeon. The working diagnosis of ISCH was established based on characteristic findings seen on axial (Ax) and sagittal (Sag) high-resolution MRI sequences (Sag T1, Sag T2, Sag T2_dixon, Sag T2_space, Ax T2 et Sag flow_flash_gradient (Phase contrast MRI)) obtained from a 1.5 tesla Magnetom Avanto (Siemens, Munich, Germany), as described in the literature [4, 5]. Strict radiologic criteria were used, considering the gold standard for this diagnosis being intra-operative. These included focal ventral displacement and sharp angulation of the spinal cord (Fig. 1) [5]. The corresponding dorsal CSF space was enlarged without the presence of an intradural lesion or spinal arachnoid webs or cysts, as excluded with CSF flow evaluations [4]. High-resolution MRI demonstrated the herniated cord, when clearly present, as subtle signal change within the ventral epidural space [5]. The exclusion criteria were patients with a history of spinal surgery or tumor (intra- and extradural) within two levels of the ISCH, infectious, congenital, or traumatic pathology with thoracic spinal cord involvement. When performed, the surgical procedure consisted in lysis of adhesions and applying an anterior dural patch, a technique previously described [13]. All were elective surgeries.

A fortuitous discovery was defined as an incidental finding on MRI, in an asymptomatic patient or whose symptoms did not clinically correlate to the abnormality found.

Asymptomatic patients were defined as having no objective neurological impairment or neuropathic pain. Patients reporting subjective symptoms, not correlated to a spinal cord origin, were also considered as asymptomatic. Patients experiencing at least one of these symptoms: Sensory and/or motor disorders, neuropathic pain, and bowel and/or bladder impairment, were defined as symptomatic.

Neurological assessments were conducted using the International Standards for Neurological Classification of Spinal Cord Injury (ISNCSCI) [14]. Sensory and motor levels, Neurological Level of Injury (NLI), sensory scores (ASIA (American Spinal cord Injury Association) pin prick and light touch score), and motor scores (ASIA Upper Extremity (UEMS) and Lower Extremity (LEMS) motor score, combined to give ASIA motor score) were determined using the ISNCSCI scale. Neuropathic pain was differentiated from musculoskeletal pain using the International Spinal Cord Injury Pain (ISCIP) Classification [15] and the International spinal cord injury Pain Basic Data Set [16]. The Brief Pain Inventory (BPI) was also used to quantify pain severity [17]. BPI scores four pain severity items and seven pain interference items rated on 0–10 scales. Higher scores indicate higher pain severity and impact on daily living activities. Bowel and bladder impairment was rated using the Autonomic standards assessment form [18].

Patients’ data were collected retrospectively on their medical charts by a trained physiatrist senior resident. The data collected included age, gender, height and weight, tobacco use, employment, comorbidities (Charlson Index [19]), possible etiological factors, number of years since diagnosis, duration between symptoms onset and diagnosis, duration of the follow-up, clinical evolution of the condition as reported by physiatrists and/or neurosurgeons, initial and follow-up clinical symptoms (including pain, sensory disturbance, motor weakness, bladder dysfunction, spasms), initial and follow-up physical exam data (including motor score of lower limbs based on MRC muscle scale, sensory level and spasticity), and the type of management (conservative or surgical). When performed at the final follow-up, data from different questionnaires were collected (Autonomic standards assessment form [18], BPI [17], International spinal cord injury Pain Basic Data Set [16] and SF-12 quality of life questionnaire [20]). For each case, MRI images were analyzed and classified by vertebral level of impairment on sagittal images by trained neuroradiologists and reviewed by a trained spine neurosurgeon.

At follow-up, clinical outcomes were classified according to changes in the motor score. Based on the minimal clinically important difference for the ASIA LEMS defined at 3.66 [21], patients were classified as “worse” if they lost 4 points or more on the ASIA LEMS, “improved” if they regained 4 points or more on the ASIA LEMS and “stable” if there were no clinically significant changes on the ASIA LEMS.

## Results

### Demographics

**Fig. 2:**
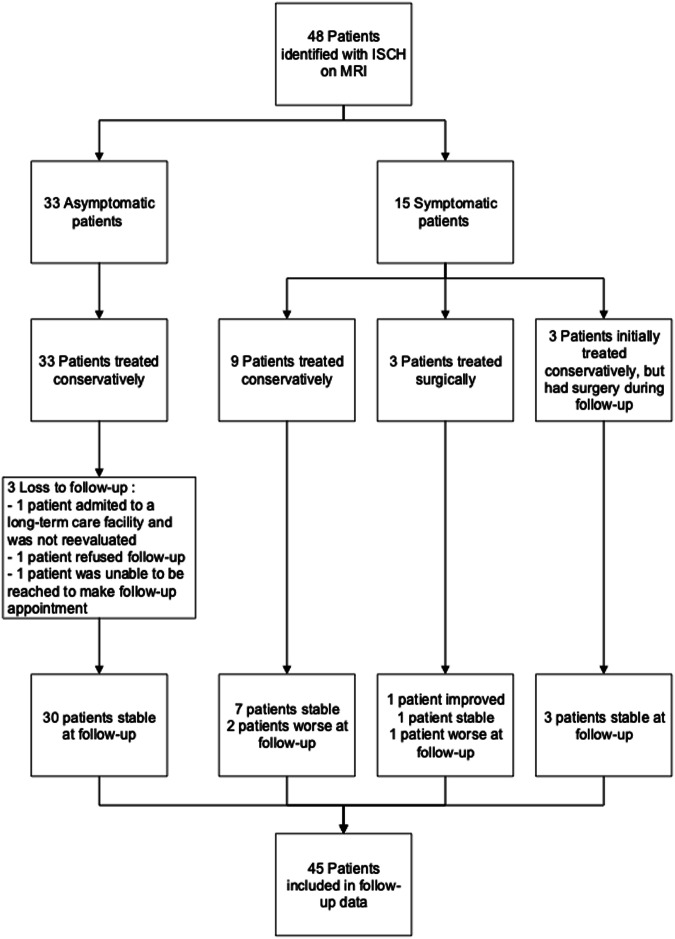
Flowchart of study participants. Among 48 patients identified with ISCH, 45 were included in the study: 30 asymptomatic and 15 symptomatic.

As shown in Fig. 2, 48 cases were identified with a working diagnosis of ISCH, between 2009 and 2019. Most of them, 33 patients, were asymptomatic (69%) and 15 patients were symptomatic (31%). Three (3) patients were unable to be reached at follow-up in the asymptomatic group.

Sociodemographic data are presented in Table 1. All variables were similar between asymptomatic and symptomatic patients. The mean age at the time of diagnosis was 52.5 years. There were as many females as males in our series with most females in the symptomatic group.

**Table 1:** Sociodemographic data of the case series.

	All patients	Asymptomatic patients	Symptomatic patients
Patients, *N* (%)	48 (100)	33 (69)	15 (31)
Age at diagnosis (Years), mean (SD)	52.5 (12.6)	52.3 (12.9)	53.1 (12.2)
Age at diagnosis (Years), median (range)	55 (47)	54 (46)	56 (45)
Gender			
Female, *N* (%)	25 (52)	16 (48)	9 (60)
Male, *N* (%)	23 (48)	19 (52)	6 (40)
Body Mass Index (kg/m^2^), mean (SD)	28.9 (7.1)^a^	29.8 (9.1)^b^	27.0 (5.9)
Smoking status, *N* (%)			
Smoker	8 (17)^a^	5 (16)^b^	3 (20)
Former smoker	13 (28)^a^	7 (23)^b^	6 (40)
Non-smoker	25 (54)^a^	19 (61)^b^	6 (40)
Charlson Comorbidity Index, mean (SD)	0.9 (1.1)	0.9 (1.1)	0.7 (0.9)
Employed, *N* (%)	27 (59)^a^	18 (58)^b^	9 (60)
Presumed etiological origin, *N* (%)			
Trauma^c^	22 (46)	16 (48)	6 (40)
Scoliosis >25°	1 (2)	0 (0)	1 (7)
Kyphosis >45°	2 (4)	2 (6)	0 (0)
Surgery of the cervical spine	1 (2)	0 (0)	1 (7)
Intradural lesion^d^	1 (2)	1 (3)	0 (0)
Meningitis	1 (2)	1 (3)	0 (0)
Unknown	20 (42)	13 (39)	7 (47)
Discovery related to symptoms, *N* (%)			
Yes	15 (31)	0 (0)	15 (100)
No	33 (69)	33 (100)	0 (0)
Spinal cord adhesion or ISCH level, *N* (%)			
T6 and above	41 (85)	28 (85)	13 (87)
Below T6	7 (15)	5 (15)	2 (13)

^a^*N* = 46.

^b^*N* = 31.

^c^Motor vehicle accident, fall from more than 1 meter, fall on back or thorax and sports-related trauma.

^d^More than two levels form the ISCH.

For both groups, a past traumatic event (22 patients, 46%) was the most frequent possible or presumed etiological origin of the pathology. The absence of any identifiable etiology was also frequent (20 patients (42%)).

The majority (85%) of spinal cord adhesion or ISCH were at level T6 or above.

### Initial clinical presentation

The initial symptoms of patients leading to the MRI investigation are reported in Table 2.

**Table 2:** Clinical characteristics.

	Asymptomatic (*N* = 33)	Symptomatic (*N* = 15)
Initial clinical presentation		
Reason for MRI, *N* (%)		
Musculoskeletal pain	18 (55)	0 (0)
Investigation for other conditions	8 (24)	0 (0)
Further investigation after cervical MRI	5 (15)	0 (0)
Trauma	2 (6)	1 (7)
Clinical myelopathy	0 (0)	8 (53)
Neuropathic pain with sensory impairment	0 (0)	2 (13)
Neuropathic pain	0 (0)	3 (20)
Sensory symptoms	0 (0)	1 (7)
Interval before diagnosis (months), mean (SD)	N/A	53.1 (61.3)
Clinical presentation, *N* (%)		
Brown-Séquard-like syndrome	N/A	8 (53)
Paraparesis	N/A	1 (7)
Sensory impairment	N/A	1 (7)
Sensory symptoms	N/A	1 (7)
Sensori-motor impairment	N/A	1 (7)
Neuropathic pain	N/A	3 (20)
Specific clinical findings		
Pain, *N* (%)		
Musculoskeletal	24 (73)	3 (20)
Neuropathic	0 (0)	9 (60)
Mixed	2 (6)	1 (7)
None	7 (21)	2 (13)
Sensory impairment, *N* (%)		
Yes	4 (12)^*a*^	10 (67)
No	29 (88)	5 (33)
Motor weakness, *N* (%)		
Yes	3 (9)^*a*^	7 (47)
No	30 (91)	8 (53)
Spasticity, *N* (%)		
Yes	0 (0)	5 (33)
No	33 (100)	10 (67)
Neurogenic bladder or bowel, *N* (%)		
Yes	1 (3)^*a*^	9 (60)
No	32 (97)	6 (40)
Management, *N* (%)		
Surgery	0 (0)	3 (20)
Conservative	33 (100)	9 (60)
Surgery during follow-up	0 (0)	3 (20)

*N/A Not Applicable*

^a^Secondary to other neurological condition (meningioma, myelitis, radiculopathy, traumatic lumbosacral plexopathy, CIDP (Chronic Inflammatory Demyelinating Polyneuropathy), polyneuropathy not yet determined).

A working diagnosis of ISCH was considered fortuitous for all patients of the asymptomatic group with musculoskeletal pain (55%), investigation in the context of another medical condition (24%) and further investigation after visualizing the defect on cervical MRI (15%) as the main reasons leading to MRI.

The symptomatic group presented mainly with clinical myelopathy (53%) or neuropathic pain, with or without sensory symptoms (33%). While the main neurological presentation was Brown-Séquard-like syndrome (53%), neuropathic pain was the second most common initial symptom (20%). The mean interval before diagnosis for symptomatic patients was 53 months, varying from 1 month to 16 years.

Initial management consisted in clinical follow-up (conservative management) or surgery. All asymptomatic patients were followed clinically. In the symptomatic group, 12 patients (80%) were initially treated conservatively. These patients did not have significant motor impairment, except one who had a unilateral weakness but was stable and was doing well functionally for many years. Three patients (20%) were treated surgically after evaluation by the neurosurgeon, mainly for significant motor impairment.

### Clinical evolution during follow-up

Initial and follow-up symptoms for asymptomatic and symptomatic patients are reported in Table 3 and Table 4, respectively.

**Table 3:** Follow-up of asymptomatic patients.

	Initial (*N* = 33)	Follow-Up (*N* = 30)
Follow-up duration in months, mean (SD)	N/A	56.1 (32.8)
Follow-up duration in months, median (range)	N/A	52.5 (131)
Pain, *N* (%)		
MSK	22 (67)	4 (13)
Neuropathic	0 (0)	0 (0)
Mixed	2 (6)	3 (10)
None	7 (21)	20 (67)
Secondary to other neurological condition	2 (6)	3 (10)
Sensory impairment, *N* (%)		
Yes	0 (0)	0 (0)
No	29 (88)	28 (93)
Secondary to other neurological condition	4 (12)	2 (7)
Motor weakness, *N* (%)		
Yes	0 (0)	0 (0)
No	30 (91)	26 (87)
Secondary to other neurological condition	3 (9)	4 (13)
Neurogenic bladder or bowel, *N* (%)		
Yes	0 (0)	0 (0)
No	32 (97)	28 (93)
Secondary to other neurological condition	1 (3)	2 (7)
Clinical outcome, *N* (%)		
Worse	N/A	0 (0)
Stable	N/A	30 (100)
Improved	N/A	0 (0)

*N/A Not Applicable*

**Table 4:** Follow-up of symptomatic patients.

	Initial (*N* = 15)	Follow-Up (*N* = 15)
Follow-up duration in months, mean (SD)	N/A	50.7 (40.3)
Follow-up duration in months, median (range)	N/A	43 (134)
Pain, *N* (%)		
MSK	3 (20)	1 (7)
Neuropathic	9 (60)	9 (60)
Intermittent Neuropathic	0 (0)	2 (13)
Mixed	1 (7)	1 (7)
Treated	0 (0)	1 (7)
None	2 (13)	1 (7)
BPI, mean (SD)		
Pain Severity Score	MD	5.0 (2.0)^a^
Pain Impact Score	MD	3.9 (2.1)^a^
SF-12, mean (difference from average)		
PCS-12	MD	33.1 (−16.9)^b^
MCS-12	MD	45.6 (−4.4)^b^
Sensory impairment, *N* (%)		
Yes	10 (67)	11 (73)
No	5 (33)	3 (20)
Secondary to other neurological condition	0 (0)	1 (7)
Motor weakness, *N* (%)		
Yes	7 (47)	8 (53)
No	8 (53)	7 (47)
Spasticity, *N* (%)		
Yes	5 (33)	6 (60)^b^
No	10 (67)	4 (40)^b^
Neurogenic bladder or bowel, *N* (%)		
Yes	9 (60)	9 (60)
No	6 (40)	6 (40)
Autonomic Standards Assessment Score, mean	MD	10/12
Clinical outcome, *N* (%)		
Worse	N/A	3 (20)
Stable	N/A	11 (73)
Improved	N/A	1 (7)

*N/A* Not Applicable, *BPI* Brief Pain Inventory, *SF-12* 12-Item Short Form Survey, *PCS* Physical Component Score, *MCS* Mental Component Score, *MD* Missing Data.

^a^*N* = 9.

^b^*N* = 10.

For asymptomatic patients, the mean follow-up duration was 56 months, varying from 12 months to 12 years. In this group, the number of patients with pain (musculoskeletal or from other neurological conditions) decreased with time and treatment. No patient in this group developed new objective sensory or motor signs upon physical examination, nor did any of them express symptoms of neurogenic bladder or bowel. Because these patients did not change their score on the LEMS, all asymptomatic patients were classified as “stable” at follow-up.

For symptomatic patients, the mean follow-up duration was 51 months, varying from 3 months to 11 years. In this group, 2 patients developed new intermittent neuropathic pain.

During follow-up, 2 patients had significant loss on the LEMS (5 and 3 points, respectively) with increased spasticity, and a third patient reported intractable neuropathic pain, secondary to an ascending syrinx. All 3 patients, initially treated conservatively, went on to have surgery. The mean follow-up period before surgical decision was 17 months, more precisely 28 months, 12 months, and 10 months for each patient. For these 3 patients, surgery stabilized the LEMS.

As noted in Fig. 2, 3 patients were considered “worse” at follow-up, losing 4 or more points on the LEMS. The first patient, treated conservatively, lost 4 points on the muscle scale from the initial assessment to follow-up. These points were lost very progressively, over multiple years. Considering this patient also had an important scoliosis and was elderly, the decision was made with the patient to have a clinical follow-up. The second one lost 5 points, but her findings and clinical evolution were difficult to interpret because she had a concomitant cervical perinatal myelopathy. In the context that she had thoracic neuropathic pain that correlated to the level of her ISCH, these new neurologic deficits in her lower extremities were also thought to be secondary to ISCH. This patient was managed medically with neuropathic pain medication and the evolution was thereafter favorable. Finally, the third patient was initially treated surgically but lost a total of 14 points at follow-up. This deterioration was slowly progressive, many years after, and not related to his surgery.

One patient treated surgically was considered “improved” after surgery as he regained 6 points on the LEMS postoperatively.

All other patients treated conservatively or surgically were considered “stable” at follow-up.

## Discussion

ISCH is a rare disease, characterized by a ventral herniation of the spinal cord through an anterior dural defect for which the pathophysiology is unclear [1–3]. ISCH cases have been more frequently reported in the past 10 years. A little more than 200 cases have been described in the literature, so far [22]. To our knowledge, our study is the case series with the largest sample of patients with a working diagnosis of ISCH. It is also the first to describe a substantial number of asymptomatic patients.

Presumed anterior spinal cord adhesion without herniation has not often been described in the literature [2, 10]. Our study reports patients with a radiological diagnosis of anterior adhesion, with or without herniation, considering the difficulty to distinguish between these two entities on radiologic factors alone. Only high-resolution T2 imaging may demonstrate the herniated cord as a subtle signal change within the ventral epidural space, which theoretically can differentiate ISCH from a simple anterior spinal cord adhesion [5]. The presence of a visible dural defect on the MRI may also be a radiologic factor helping to differentiate adhesion from herniation, but the absence of a dural defect on the imaging does not exclude the presence of herniation [2]. Taylor et al. have proposed that the association of clinical thoracic myelopathy symptoms with an anteriorly displaced and thinned thoracic cord, with or without radiological or surgical evidence of cord herniation, should be considered as a spectrum of the same disorder (thoracic anterior spinal cord adhesion syndrome) [2]. Although we believe that there may be a radiological continuum between these two entities, further analyses of patients’ radiological characteristics are needed to confirm this hypothesis and to identify possible radiologic factors contributing to the development of ISCH. We also hypothesize that the presence of symptoms may be related to the stage of the pathologic process, that can eventually lead to herniation, but other factors may also contribute, such as the angle of the spinal cord herniation and the anatomical level, as well as the midthoracic region being a watershed area.

The particularity of our sample is the high number of asymptomatic patients in which the anomaly was discovered fortuitously. The great majority of the reviews in the literature only discuss symptomatic patients [7, 8, 11, 22]. With the incidental findings in asymptomatic patients, we are faced with a difficult decision regarding their treatment plan, with an initial surgical management having too much of a high risk [23]. Therefore, clinicians in our institution opted for a conservative treatment with clinical follow-up. With patients followed-up for an average of 56 months, we showed that pathological findings do not seem to progress with neurological symptoms.

The surgical decision was primarily based on the presence of significant motor weakness, functional impairment and, after discussion about the benefits and risks with the patient. Because of the risk of neurologic deterioration with the procedure, surgery was considered too much of a risk for patients with pain or with sensory impairment only. This explains that most of our symptomatic patients were treated conservatively, three were initially treated with surgery and three had surgery during follow-up.

Clinical observation of patients is reported in the literature by only a handful of authors for follow-ups ranging from 3 months to 8 years [4, 10–12]. In our study, 39 patients (asymptomatic and symptomatic) were followed-up clinically, which makes it the largest group reported to date, with a follow-up ranging from 3 months to 12 years.

Most of our patients (41 out of 45) were considered stable or unchanged at follow-up. All asymptomatic patients demonstrated no neurological progression. In the symptomatic group, 66% of patients who had a surgical intervention, initially or during follow-up, were stabilized by surgery and one had improvement (17%). The surgical approach in our center is adapted to the intra-operative findings. It usually consists of a posterior bilateral approach with lysis of adhesions and anterior dural patch. In addition, if there is a true herniation, the dural defect is widened, and the hernia reduced. The optimal surgical technique is still unknown due to the rarity and variability of surgical procedures for this specific entity in the literature, but we believe that multiple factors should be considered, including intra-operative findings.

Our study has some limitations, as it is based on routinely collected data. Some initial data are missing with respect to the level of documentation and accurate record keeping of patients. The presumed etiology of ISCH is not confirmed considering that the time of the abnormality onset is unknown. Another limitation is the absence of correlation between MRI and intra-operative findings. Since most of our patients did not have surgery, ISCH remains a working diagnosis. Moreover, the continuum between spinal cord adhesion and herniation remains hypothetical. Nonetheless, only four out of six of our surgical patients demonstrated herniation intra-operatively, supporting this hypothesis. For this reason, the term “thoracic spinal cord tethering” or “thoracic anterior spinal cord adhesion syndrome” as proposed by Taylor and al. (2012) [2] may be more appropriate to include the spectrum of this pathology. Because the radiologic differences between adhesion and herniation are difficult to establish, considering few true radiologic factors are described, we may have overestimated the presence of a true herniation. This would also have been the case in other studies relying on a radiologic diagnosis. An extensive analysis of radiologic factors was not performed within the framework of this study, but further analyses of these factors will be carried out in a second study. This case series is mostly descriptive, limiting the interpretation of our data. Despite these limitations, this case series led to the identification of a great number of asymptomatic patients and to the evocation of preliminary data on these types of patients. This study also permitted to describe the clinical evolution of symptomatic and asymptomatic patients, with a significant follow-up duration.

In summary, idiopathic spinal cord herniation is a rare disease that is getting more attention with the increasing quality and availability of MRI. It is part of the slowly progressive myelopathy differential diagnosis. Our study showed that ISCH and its possible variants are not always symptomatic and may be a fortuitous finding. The natural history seems to be nonprogressive in most cases, especially in the asymptomatic patients. Because of this, it is probably appropriate to treat such cases expectantly.

## Supplementary information

Supplementary Table 1 Legend

Supplementary Table 1

## Data Availability

The data that support the findings of this study are included in the published article and its Supplementary information file. The individual data from each case included in this study is available from the corresponding author, upon request.
