# Supplementary material for: The clinical evolution of patients with idiopathic spinal cord herniation: a case series
Source: Spinal Cord Ser Cases. 2024 Oct 9;10:71. doi: 10.1038/s41394-024-00684-9 (PMC11464882; doi:10.1038/s41394-024-00684-9)
Supplement: Supplementary file 1 — Supplementary Table 1 Legend [file 41394_2024_684_MOESM1_ESM.docx]

Supplementary Table 1 Detailed Neurologic Examination and Evolution of Symptomatic Patients.

*^a^* Inconsistent; *^b^* Perinatal Myelopathy; *FU, Follow-up; R, Right; L, Left*
